# Multiscale Elasticity of Epoxy Networks by Rheology and Brillouin Light Spectroscopy

**DOI:** 10.1021/acs.jpcb.4c06492

**Published:** 2024-12-04

**Authors:** Emmanouela Filippidi, Anuj K. Dhiman, Benke Li, Thanasis Athanasiou, Dimitris Vlassopoulos, George Fytas

**Affiliations:** † Department of Materials Science and Engineering, University of Crete, Heraklion 70013, Greece; ‡ Institute of Electronic Structure and Laser, FORTH, Heraklion 70013, Greece; § Faculty of Physics, Adam Mickiewicz University, Uniwersytetu Poznanskiego 2, Poznan 61614, Poland; ∥ Max Planck Institute for Polymer Research, Ackermannweg 10, Mainz 55128, Germany

## Abstract

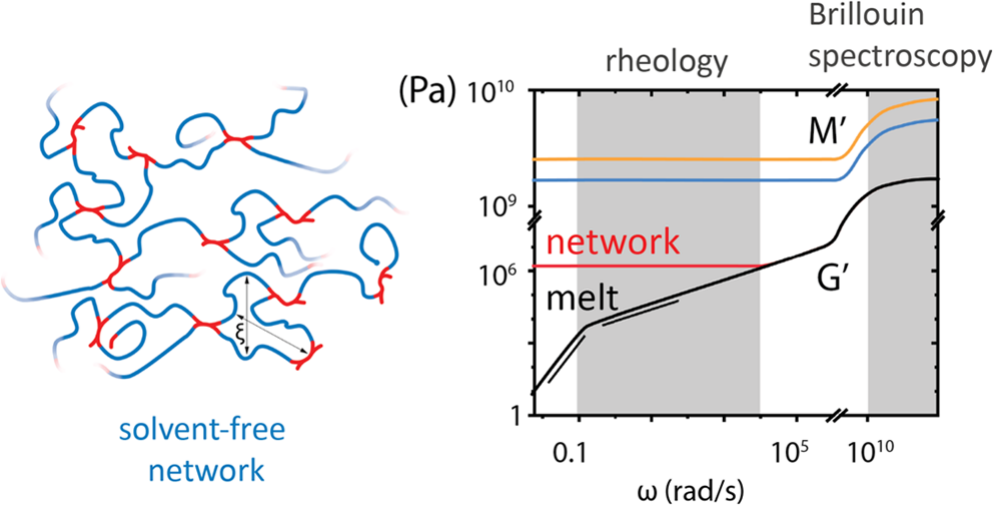

The response of soft materials to an imposed oscillatory stress is typically frequency dependent, with the most utilized frequency range falling in the range of 10^–2^–10^2^ rad/s. In contrast to most conventional contact techniques for measuring material elasticity, like tensile or shear rheology and atomic force microscopy, or invasive techniques using probes, such as microrheology, Brillouin light spectroscopy (BLS) offers an optical, noncontact, label-free, submicron resolution and three-dimensional (3D) mapping approach to access the mechanical moduli at GHz frequencies. Currently, the correlation between the experimental viscoelastic (at lower frequencies) and elastic (at higher frequencies) moduli has fundamental and practical relevance, but remains unclear. We utilize a series of solvent-free epoxy polymer networks with variable cross-link density as models to compare the storage modulus, *G*′, (in the MPa range) obtained from shear rheology and the longitudinal modulus, *M*′, (in the GPa range) extracted from BLS. Our results show that *G*′ exhibits a much stronger increase with increasing cross-link density than *M*′ (by a factor of about 3.5). This finding is discussed in the context of the phantom network model for *G*′ and Wood’s inverse rule of mixtures for *M*′. The epoxy polymer network displays an unexpectedly fast hypersonic dispersion compared to its uncross-linked precursor. These results testify the importance of obtaining reliable information about the elasticity of networks and will hopefully trigger further investigations in the direction of bridging the elasticity of soft materials at different scales.

## Introduction

Tailoring material elasticity is a formidable challenge for the design and production of a wide range of materials, including foodstuff, personal care products, drug formulations, but also for the understanding of biological materials, cells^1,2^ and tissues. For example, the local stress in cells may alter gene expression^2^ demarcating health and disease. The dynamic response of soft materials is often frequency dependent, covering a broad range of moduli, from Pa at low frequencies (which can typically reach 10^–2^ rad/s) to GPa at high frequencies^3^ (which can exceed 10^3^ rad/s). Material elasticity is typically characterized by moduli such as Young’s modulus *E*, storage and loss shear moduli *G*′ and *G*″, respectively, bulk modulus *K*, which traditionally require contact forces to be measured, e.g., shear or extensional rheology^4,5^ and atomic force microscopy.^6^ An exception is the noncontact, label-free, all-optical, microscopic measurement of the longitudinal modulus, *M*, via Brillouin light spectroscopy (BLS), which allows for three-dimensional (3D) elasticity mapping and has attracted renewed interest^7−12^ in various fields of materials science, including biomedical applications. It should be noted, however, that BLS-based measurement of *M*, and sometimes *G*, is performed at the GHz frequency range, much higher than the conventional (and most utilized) regime of soft materials. While the low-frequency viscoelastic moduli are well understood in the framework of polymer viscoelasticity, leading to derivations of scaling laws for polymer dynamics,^3^ or phenomenologically described with Arrhenius and non-Arrhenius models for glass-forming liquids, there is no conceptual framework to describe the local nature of the elastic moduli at high frequencies. This prevents the fundamental understanding of the complete broadband mechanical spectra of different materials,^13^ but also the robust extraction of low-frequency *G*, based on high frequency BLS measurements of *M*. Several^13,14^ experimental studies focused on the glass forming liquids by measuring an impressive range of 13 decades in frequency with various techniques, while efforts were undertaken to understand theoretically^15^ and phenomenologically^8^ the link between high and low frequency measurements in soft materials, such as hydrogels. For polyacrylamide hydrogels^8^ a correlation was proposed, based on the conformity of the real part of the longitudinal modulus *M*′ to an inverse rule of mixtures, and of the storage shear modulus *G*′ to a scaling relation for polymer networks based on the effective polymer volume fraction and the cross-link density. This material-dependent correlation was less successful when the hydrogels were swollen to equilibrium due to a swelling ratio dependent scaling. The much stronger variation of *G*′ with the cross-link density compared to that of *M*′, that renders their much-desired correlation material-dependent, is our motivation to extend that work to solvent-free polymer gels.

**Figure 1:**
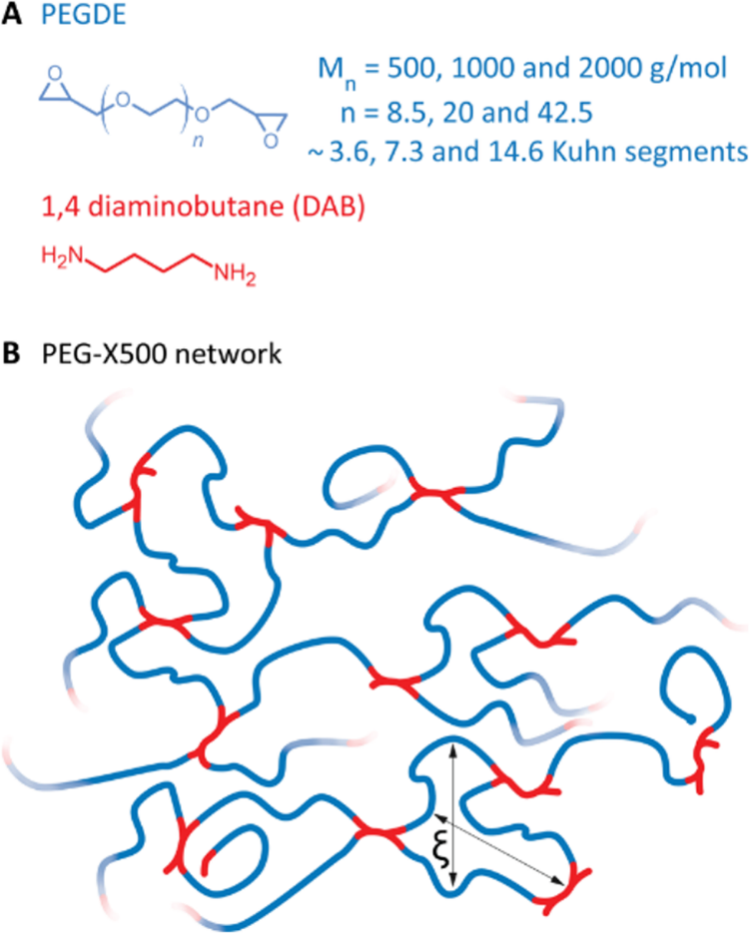
(A) Networks are formed by reacting diepoxide PEGDE polymer with diamine DAB cross-linker. PEGDE of three different molecular masses has been used to create networks with three different cross-link densities. (B) Molecularly faithful in two dimensions PEG-X500 network of PEGDE-500 and DAB, where ξ denotes the mesh size.

In this work, we focus on the properties of epoxy polymer networks, which are ubiquitous in applications such as lightweight materials, efficient energy-absorbing materials or adhesives. They cover an intermediate stiffness regime compared to the aforementioned hydrogels and molecular glasses, and their curing kinetics have been studied in both the low^16^ and the high frequency^4^ regimes. In particular, we examine networks based on a bifunctional, telechelic linear diepoxide with variable average molar mass (500, 1000, and 2000 g/mol) cross-linked via a flexible tetrafunctional diamine cross-linker (Figure 1). Varying the molar mass of the poly(ethylene glycol) (PEG)-based PEGDE oligomer from 500 g/mol ≅ *M*_e_/4 to 2000 g/mol ≅ *M*_e_, where *M*_e_ ≈ 2000 g/mol is the entanglement molar mass of linear PEG, yields networks ranging from amorphous (500 g/mol) to semicrystalline^4^ (Table S1 and Figure S1). In this way, we prevent entanglements at the lowest molar mass and highest cross-link density and barely reach the entanglement limit at the highest molar mass and lowest cross-link density. Equivalently, by varying the number of Kuhn segments of the PEGDE oligomeric precursor from 4 to 15 (Kuhn molar mass is 137 g/mol^3^) we reach the lowest limit of applicability of the Gaussian chain model. Finally, our networks are solvent-free. By measuring the shear moduli *G*′, *G*″ with rheology, and the longitudinal *M*′, *M*″ with BLS, we complete the mechanical response of the network over broad frequency range over broad frequency range, examine the sensitivity of BLS to changes in cross-link density and assess the correlation between the low and high-frequency response of the networks. Such correlations are not only of interest to experimentalists to expand to noncontact applications in biomedicine or to connect low and high frequency mechanical responses, but are also important in the quest to compare atomistic simulation results on elastic constants at high frequencies, typically at extensional rates of 10^8^–10^9^ 1/s,^17^ against experimental data at lower frequencies. More generally, understanding of the multiscale elasticity of polymeric networks is necessary for the design of new systems with desired performance.

## Materials and Methods

### Network Formation

Telechelic epoxide functionalized poly(ethylene glycol) diglycidyl ether (PEGDE, CAS 26403–72–5) with number-average molar masses, *M*_n_ = 500, 1000, and 2000 g/mol, of dispersity 1.38 for PEDGE-500, 1.69 for PEGDE-1k and 1.09 for PEGDE-2k as measured by gel permeation chromatography (GPC) in tetrahydrofuran (THF) (Agilent II, mixed-C, mixed-D and mixed-E PLgel columns in series, calculation based on refractive index trace) and tetrafunctional 1,4-diaminobutane (DAB, CAS 110–60–1) of 88.15 g/mol were purchased from Sigma-Aldrich. PEGDE with *M*_n_ = 1000 g/mol was purchased from PolySciences Inc. The number of repeat units was also measured with solution proton nuclear magnetic resonance (^1^H NMR) and were determined to be 8.1, 23.2, and 51.5 respectively for PEGDE-500, 1 and 2k. Thus, PEGDE-2k’s calculated *M*_n_ = 2469 g/mol. PEGDEs and DAB were individually brought to the liquid state well before mixing. Epoxy networks were synthesized per compositions of Table S1 by mixing PEGDE and DAB stoichiometrically in terms of functional groups. The diamine contains two primary amines, each of which can react twice with each epoxide (Figure 1). The mixture was vortexed, then degassed for 10 min, at a pressure below 1 Torr, poured and cured between poly(tetrafluoroethylene) (PTFE) slides with a 0.5 or 0.25 mm spacer at 60 °C and 600 Torr for 36 h. After removal of the slides, the samples were kept in vacuum at room temperature until measuring. Densities were extracted from mass weighing and volume estimation from the thickness and area using a digital micrometer and imaging, respectively. For PEG-X500 the density was 1.15 ± 0.01 g/cm^3^ and for the other two networks 1.10 ± 0.01 g/cm^3^ .

**Figure 2:**
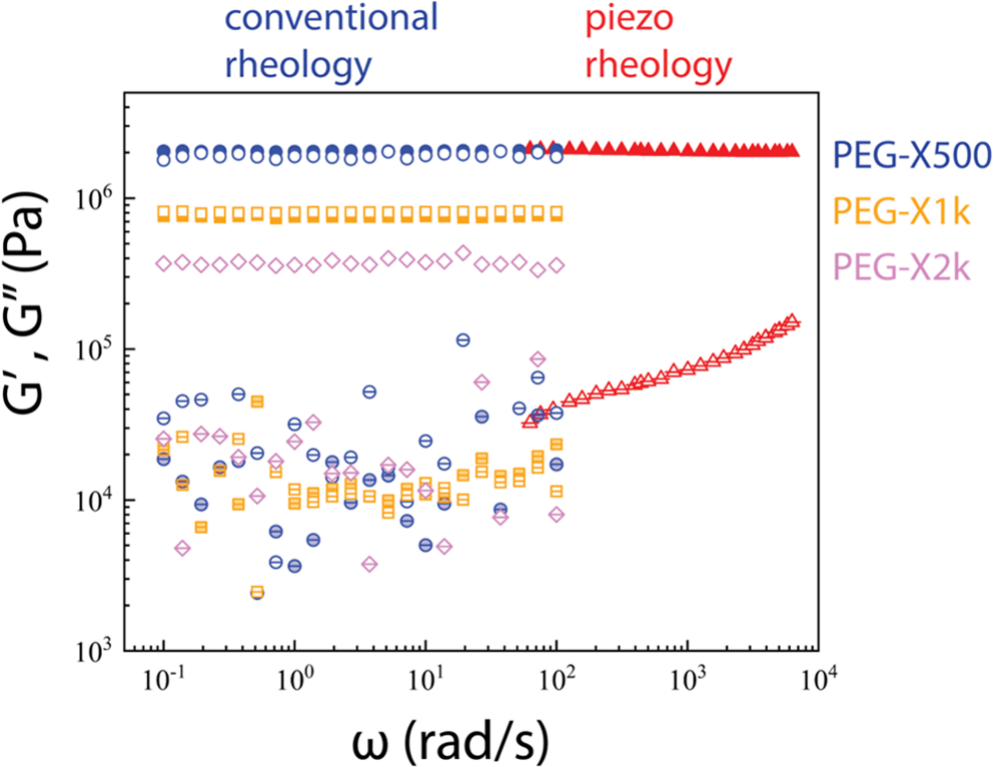
Dynamic shear moduli of PEG-X500 (blue and red), PEG-X1k (orange) and PEG-X2k (purple) at 25 °C (closed symbols) and 65 °C (open symbols) combining conventional (blue, orange, purple symbols) and piezoelectric high-frequency rheology (red triangles). PEG-X2k is semicrystalline at room temperature, so data is only shown at 65 °C. *G″* is shown as symbols with inner horizontal line. *G′* as fully empty or filled symbols.

### Differential Scanning Calorimetry (DSC)

DSC (TA Instruments DSC 250) was initialized with an isothermal jump at 110 °C for 2 min to remove humidity, followed by two full cooling–heating cycles in the range of −100 to 80 °C with a heating and cooling rate of 10 °C/min. The resulting scans of the two cycles were identical. From the DSC traces (Figure S1) the glass transition and melting temperatures, *T*_g_, and *T*_m_ respectively, were determined (Table S1).

### Abbe Refractometry

Refractive index measurements of the precursors were performed on a J457 Refractometer (Rudolph Research Analytical) equipped with sodium-D line (598 nm) and temperature control (Table S2).

### Linear Viscoelasticity (LVE)

Small amplitude oscillatory shear measurements of the networks (Figure 2) were performed with a strain-controlled rotational rheometer ARES 2kFRTN1 (TA) with stainless steel parallel plates (4 mm diameter, 0.45 mm gap). Sandpaper of 320 grit was carefully glued to the plates to prevent wall slip of the network films and plate parallelity was confirmed. Strain amplitude of 0.1% in the linear regime was applied. Temperature control of ±0.1 °C was achieved with a convection oven fed with nitrogen gas to create an inert atmosphere. Samples were measured at 25 and 65 °C. To ensure complete crystal melting, PEG-X2k was annealed at 85 °C for 15 min before let to equilibrate for an additional 15 min at the measurement temperature of 65 °C. The frequency spectrum obtained by the commercial rotational rheometer (10^–1^–10^2^ rad/s) was extended to higher frequencies (reaching 7 × 10^3^ rad/s) by using a homemade high frequency piezo-rheometer (PZR).^5,18,19^ In this strain-controlled rheometer, a voltage waveform excites the upper piezo actuator to induce a proportional strain, γ(*t*), while the resulting stress is probed by the lower piezo ceramic which acts as a shear stress sensor via the proportional generated electric charge. Charge is translated into a voltage waveform by means of a charge amplifier. The two signals corresponding to strain excitation and stress response are correlated in a lock-in amplifier where phase angle and modulus magnitude are extracted. The setup was calibrated with high-frequency reference data of a polybutadiene melt, obtained by time–temperature superposition.^18^ As a very thin specimen is needed for high-frequency rheology, a 200 μm-thick PEG-X500 film was prepared. A disc with diameter of 8 mm was punched from the film. The stiffness of the piezoelectric ceramics and the small applied strain amplitude (10^–5^%) make PZR ideal to probe this polymer at the cross-linked state. Note that the high frequency regime can be probed by passive microrheology as well,^20,21^ however this technique requires the use of large nonadsorbing probes which can be questionable in heterogeneous samples such as the present ones, hence the PZR method is preferred.

### Brillouin Light Spectroscopy (BLS)

**Figure 3:**
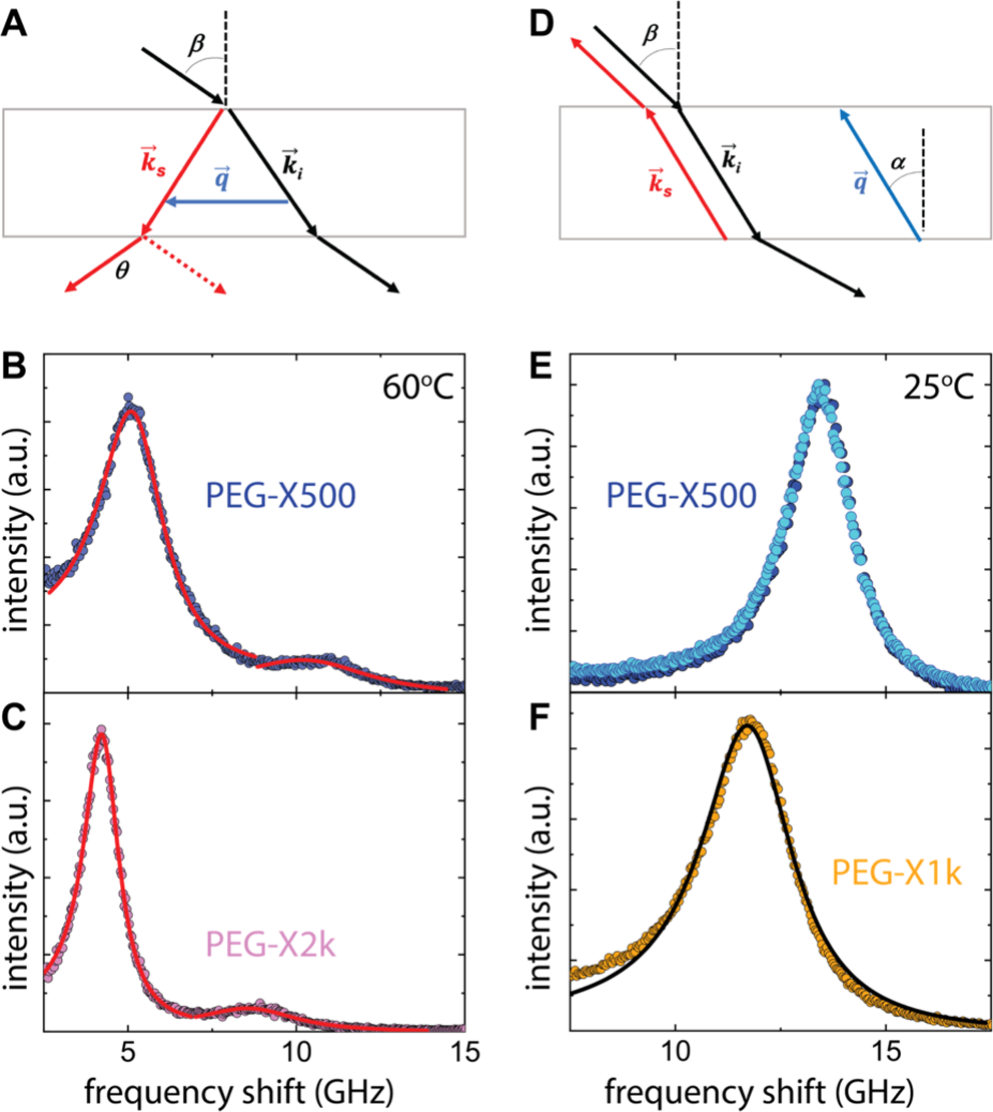
BLS spectra (anti-Stokes frequency side). Schematic light paths for transmission (A) and backscattering (D) geometries. (B, C and E, F) Collected BLS spectra (circles) and fits to the damped harmonic oscillator model, eq 1 (solid lines). (B, C) BLS spectra recorded at transmission geometry (θ = 90°, *q*_**||**_ = 0.0167 nm^–1^) and 60 °C of (B) PEG-X500 and (C) PEG-X2k. The second, high frequency peak is the artificial backscattering from the reflected light at the exit side of the film. (E, F) BLS spectra recorded at backscattering (θ = 180°, *q*_bs_ = 0.035 nm^–1^) and 25 °C of (E) PEG-X500 where two samples (dark blue and cyan symbols) prepared under the same conditions are considered and (F) PEG-X1k.

BLS was employed at transmission (Figure 3A) and backscattering (Figure 3B) geometries at room temperature (25 °C) and 60 °C at wavelength λ = 532 nm (in vacuum) at about 50 mW laser power using a tandem Fabry–Perot interferometer (Table Stable Ltd., Switzerland). For the transmission geometry with incidence angle β and scattering angle θ = 2β, the scattering wavevector, *q⃗*_||_ = *k⃗*_s_ – *k⃗*_i_, is parallel to the film surface and has a magnitude *q*_||_ = 2 *k*_i_ sin(β) that is independent of the medium refractive index *n*. *k⃗*_i_ and *k⃗*_s_ are the incident and scattered light wavevectors, respectively. We obtain the longitudinal sound velocity, *c*(*q*) = ω_B_/*q*, directly from the frequency shift, ω_B_, in the range (5–100) × 10^9^ rad/s, without knowledge of *n*. For the backscattering (θ = 180°) geometry, the magnitude *q*_bs_ = 2*k*_i_*n* assumes its highest value, corresponding to the highest possible frequency, ω_bs_. The BLS spectrum of density fluctuations is represented by the damped harmonic oscillator
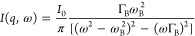
1where ω_B_ is the frequency and Γ_B_ the half-line width (HWHM) of the Brillouin peaks. The storage and loss moduli are obtained from
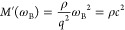
2a
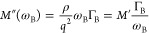
2bwhere ρ is the mass density of the material. The longitudinal modulus is M* = *M*′ + *i M*″ = K* (ω) + 4/3 G* (ω). The relationship between K* and G* is determined by the Poisson ratio, ν, which controls the contribution of the shear modulus at high frequencies .

## Results and Discussion

### Low-Frequency Regime: Shear Modulus from LVE

The measured dynamic storage, *G*′, and loss, *G*″, moduli of the cross-linked networks PEG-X500, PEG-X1k and PEG-X2k via conventional oscillatory rheology, complemented by piezo-rheometry for PEG-X500, are shown in Figure 2. For the amorphous PEG-X500 and PEG-X1k networks, *G*′ is virtually temperature-independent between 30 and 65 °C. All data indicate frequency-independent *G′* over several decades (5 for PEG-X500 and 3 for the rest) and nearly frequency independent *G*″ with a weak increase with increasing frequency for PEG-X500 at high frequencies (with a slope of about 0.2). The *G*″ data are quite scattered in the 10^–1^–10^2^ rad/s regime of conventional rheometry. This is likely due to fluctuations in the stress signal associated with the low phase angle and has been reproduced by repeated measurements. It could be improved by longer measurements per frequency and larger strain amplitudes in the linear viscoelastic regime, but this has not been attempted since the overall picture is unambiguous, especially the *G*′ signal which is of prime interest here. Note that the examined networks are heterogeneous, as also discussed in a recent publication which focused on PEG-X500 and PEG-X1k.^4^ Qualitatively, for identical stoichiometry, increasing the PEGDE precursor molar mass leads to decreased modulus, since there is an equivalent decrease in cross-link density. For the semicrystalline network PEG-X2k at 30 °C < *T*_m_, *G*′ ≈ 6.7 MPa, but at 65 °C > *T*_m_, its value drops below that of PEG-X1k, as expected. Numerical values are shown in Table 1.

**Table 1:** Mechanical Properties of the Networks

network	*G*′_measured_ at 65 °C (MPa)	*G*′_phantom_ (MPa)	*M*_c,phantom_ (g/mol)	*f*_phantom_	*M*′ at 25 °C (GPa)
PEG-X500	2.03 ± 0.02	2.54 ± 0.07	740 ± 20	2.92	6.5 ± 0.2
PEG-X1k	0.75 ± 0.05	1.31 ± 0.03	1900 ± 50	2.28	4.6 ± 0.2
PEG-X2k	0.36 ± 0.02	0.68 ± 0.02	3970 ± 100	2.12	4.2^a^

a Estimated for an equivalent amorphous network at this temperature (see text).

Analysis with the phantom network model,^3^ which allows for fluctuations of network junctions, predicts a plateau modulus , where *f* = 4 is the expected functionality of the diamine cross-linker, *R* the ideal gas constant, *T* the absolute temperature and *M*_c,phantom_ the average molar mass of the chain between cross-links. From the sum of the PEGDE and DAB molar masses, we estimate a minimum *M*_c,phantom_ = 588, 1088, and 2088 g/mol for the three networks, respectively. Using the measured density, ρ, the previously measured epoxide conversion^4^ of 93 ± 2%, which scales the functionality to 0.93*f*, and either (1) *M*_c,phantom_ = *M*_n_ of PEGDE, or (2) the measured plateau value of *G′* (Figure 2) or (3) both, we respectively calculate the *G′*_phantom_ and *M*_c,phantom_ or *f*_phantom_, which are presented in Table 1. Clearly, the phantom network model provides the correct qualitative picture but does not describe quantitatively the different networks. Note that the predictions are better (close to quantitative) for PEG-X500 and deviate as the molar mass increases. Adjusting the calculations for the NMR-determined *M_n_* of PEGDE-2k does not provide quantitative agreement either, as the *G′*_phantom_ only decreases by 10%. In particular, values of *f*_phantom_ < 3 are incommensurate with the existence of percolating networks which we certainly form, as the PEG-X networks can all be successfully swollen and redried without identifiable change in moduli. Other reasonable factors explaining the discrepancy include precursor polydispersity in the commercial polymer, the presence of dangling ends,^4^ and spatial heterogeneity. Based on revised theories of network elasticity,^22,23^ our previous work^4^ concluded that the contribution of dangling ends, rather than the presence of loops, is at the origin of the deviation from the phantom model. Regarding spatial heterogeneity, a topic extensively studied in various epoxy networks^4,24−26^ whose glass transition temperature, *T*_g_, reaches the curing temperature,^27^ the formation of stiffer “nodules” of tens of nanometers during polymerization has been observed. They are thought to be localized areas of fast, early polymerization during the curing process resulting in stiffer nodules surrounded by a softer matrix. As the PEG-X500 and PEG-X1k networks’ curing temperature of 65 °C remains significantly above *T*_g_ at all times, we do not expect such a nodule formation here. Small-angle X-ray scattering (SAXS) data, shown in Figure S2 do not support any structure formation for the PEG-X500 network. The low-q contribution (often referred to as small angle upturn) indicates the presence of inhomogeneities at length scales larger than 30 nm. For the PEG-X1k network, only a shoulder related to emerging crystallinity is evident. This is absent in the fully amorphous PEG-X500 networks. Despite these issues, a notable outcome of Table 1 is the ratio of *M*_c,phantom_ values of the three networks PEG-X500/PEG-X1k/PEG-X2k, which is nearly identical to that obtained from chemistry (above). This suggests a consistency of the data and similarity of the network structures. The consideration of similar network structure is based on the same chemistry and the average values of Mc extracted from the phantom network model. While this is a reasonable assumption, some deficiencies and related heterogeneities cannot be excluded, as already mentioned. Application of ^1^H double-quantum NMR spectroscopy^28,29^ could possibly reveal quantitative, comparative details among the epoxy networks regarding their heterogeneous microstructure, the content of dangling chains and cross-link density.

### High-Frequency Regime: Longitudinal Modulus from BLS

Based on theoretical predictions,^15^ and earlier experiments^8−12^ the sound waves should interfere with the density fluctuations arising from relatively fixed local network configurations at the cross-links,^30^ which differ from those of the connecting PEG network strands and the freely fluctuating dangling ends. We contrast un-cross-linked PEGDE of three molecular masses against cross-linked networks based on the same PEGDE. The uncross-linked PEG volume fraction increases with increasing PEGDE molar mass from 500 to 2000 g/mol. Experimentally, to avoid the interference of crystallization of the PEG-X2k, we measured all three cross-linked films at 60 °C in transmission, and PEG-X500 and PEG-X1k at 25 °C in backscattering (Figures 3 and S3). The BLS spectra of PEG- X500 and PEG-X2k display single longitudinal phonon peaks separated by about 0.8 GHz at the same *q*_||_ = 0.0167 nm^–1^. At 60 °C, the frequency difference between PEG-X500 and PEG-X1k amounts to about 0.7 GHz (Figure S3) and it becomes just about 0.1 GHz between PEG-X1k and PEG-X2k, as the cross-link density decreases with increasing PEG molar mass. The second, high frequency peak at all transmission spectra is the artificial backscattering from the reflected light at the exit side of the film. That leads to an estimate of the refractive index, *n* ≈ 1.48, in reasonable agreement with the refractometer data of the melts (Table S2). Solid lines in Figure 3 indicate the representation of the experimental BLS spectra by the damped harmonic oscillator model (eq 1), that yields the longitudinal sound velocity *c* of PEG-X500, PEG-X1k and PEG-X2k of 1910 ± 20, 1660 ± 20 and 1580 ± 15 m/s, respectively. The cross-linked films PEG-X500 and PEG-X1k are amorphous at 25 °C, and their BLS spectra are shown in Figure 3E,F at the highest *q*_BS_ = 0.0354 nm^–1^. The value of the refractive index (1.48) needed to compute *q*_bs_ was obtained from the comparison with the longitudinal *f*_B_ (=ω_B_/2π) of the BLS spectra recorded at *q*_||_ = 0.0167 nm^–1^ (Figure S4). These measurements also reveal higher frequency for PEG-X500, leading to *c* = 2370 ± 25 m/s for PEG-X500 versus 2050 ± 20 m/s for PEG-X1k. Taking also into account the densities of the networks, we obtain the respective *M′* values (presented in Table 1) of 6.5 ± 0.2 and 4.6 ± 0.2 GPa, respectively. Their ratio is 1.4. Assuming the same temperature dependence *c*(T) for PEG-X2k and PEG-X1k, justified by their similar density values, we estimate *c* = 1960 m/s and *M′* = 4.2 GPa (Table 1) for the least cross-linked PEG-X2k, considering however an equivalent amorphous network at 25 °C. It is important to note that this temperature is below *T*_m_ = 37 °C (Table S1), hence the extracted *M′* value for PEG-X2k is quite uncertain. With this in mind, the number of Table 1 indicate that the most cross-linked PEG-X500 is stronger than PEG-X1k by about 40%, whereas the difference decreases (to about 10%) between PEG-X1k and PEG-X2k. This is not unanticipated, given the above note and the increasing structural similarity of the cross-linked PEG to the PEGDE precursor when the molar mass increases.

**Figure 4:**
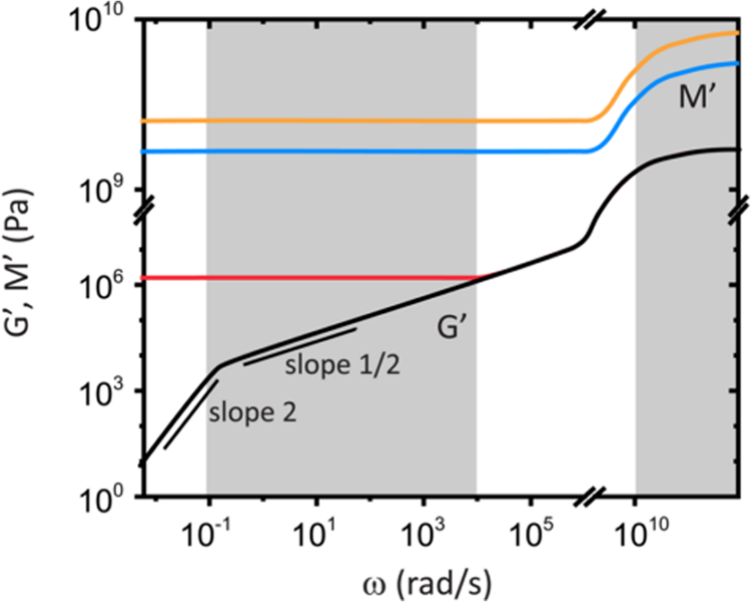
Schematic illustration of the broadband frequency dependence of longitudinal *M*′ (blue and orange) and shear *G*′ (black and red) moduli of an unentangled polymer melt (blue/black) and its cross-linked (orange/red) network analogue at *T* ≫ *T*_g_. For the melt, the scalings *G*′ ∼ ω^2^ and *G*′ ∼ ω^1/2^ refer to the first Rouse mode, the terminal relaxation of the chain at low frequencies, and viscoelastic regime (second Rouse mode) at intermediate frequencies, respectively. Gray shaded areas denote the experimental frequency range for *G*′(ω) and *M*′(ω) at low and high frequencies, respectively. The segmental relaxation frequency is roughly at the onset of the high-frequency gray zone.

The strong effect of cross-linking on the PEG elasticity can be also examined with reference to the viscoelasticity of the oligomeric PEGDE precursors. At 60 °C, where all three PEGDE precursors are viscoelastic liquids, they display very similar sound velocity (*c* = 1530 ± 15 m/s, Figure S5). This finding is rather unexpected, as the segmental free volume decreases with increasing molecular weight due to diminishing fraction of chain ends. Hence, the increased *M*′ in the cross-linked PEG should relate to the cross-linking density, as the sound velocity ratio of cross-linked PEG-X network to precursor PEGDE decreases from PEG-X500 (1.29) to PEG-X2k (1.03). However, M*(ω_B_) is frequency dependent, assuming a low value *M*_0_ (viscous, liquid-like) at low frequencies (ω_B_τ ≪ 1) and high value *M*_∞_ (elastic, solid-like) at high (ω_B_τ ≫ 1) frequencies compared to the segmental relaxation frequency (τ^–1^), as schematically shown in Figure 4. For uncross-linked amorphous polymers, the dispersion of *M′*(ω) (blue line in Figure 4) at GHz frequencies usually occurs at *T* ≈ *T*_g_ + 120 °C, as τ (*T*) relates to *T*_g_. For a Poisson ratio ν ≈ 0.4, *M*′/*G*′ *≈* 6, *K*′ *≈* 14/3 *G*′, we thus conclude that *K*′ (ω) is the primary contributor to *M*′(ω).

At low (kHz) frequencies, the dispersion M*(ω) is due to the shear G*(ω), since *K*′(ωτ ≪ 1) ≈ *K*_0_, the limiting low-frequency bulk modulus. However, *K*_0_ ≫ *G*′ and hence *M*′ is frequency independent at low frequencies (blue and orange lines in Figure 4). It should be noted that the dispersion M*(ω) can be shifted to low frequencies by increasing τ(*T*) which can be achieved by decreasing the temperature closer to *T*_g_.^13^ The shear modulus, *G*′ for unentangled chains (black line in Figure 4) exhibits terminal response at very low frequencies *G*′ ∼ ω^2^ (ωτ_R_ < 1, where τ_R_ is the Rouse time of the chain), while at intermediate frequencies, ωτ_R_ > 1 it follows *G*′ ∼ ω^1/2^. At even higher frequencies, ω_B_τ ≃ 1, *G*′ increases to reach its limiting high-frequency value (glass regime) determined by the Poisson ratio. In the case of cross-linked polymers, an increase of *M*′(ω) (orange line in Figure 4) is anticipated for the network, while the impact of cross-linking on the local relaxation cannot be a priori predicted. On the other hand, cross-linking prohibits flow at low frequencies ωτ_R_ < 1 and this regime is characterized by a plateau value for *G′* (red line in Figure 4). Hence, cross-linking should impact *G*′ more significantly than *M′*. For an unambiguous comparison of *M*′ among the PEG films, it is therefore important to know whether M*(ω) exhibits a dispersion at GHz frequencies at ambient temperature, while G*(ω) is accessible at low frequencies by shear rheology.

In earlier BLS reports of amorphous polymers^31−33^ and epoxy^16,34,35^ networks, the experiments were performed at a single scattering angle (and hence *q*) either at various temperatures well above *T*_g_ or/and at different curing times to reach ω_B_τ (*T*) ≈ 1. The temperature dependence of *M*″(T) was analyzed assuming either a relaxation time distribution or a known τ(T) for single relaxation time.^33,36^ The relaxation time τ, extracted from BLS of amorphous polymers, was found to be faster than the α-relaxation time τ_α_.^36,37^ In fact, the α-relaxation is not the only mechanism that contributes to the M*(ω) at hypersonic frequencies. This phenomenology is based only on a single *q* (frequency), but also for a few cases on data from inelastic X-rays or a ultraviolet (UV) light source.^38^ Instead, the necessary record of the isothermal dispersion, ω_B_(*q*), has rarely been the case^14,39^ on the account of only a few angular dependent BLS facilities worldwide. While for two (polyisoprene, polypropylene) amorphous polymers, τ is more than 1 order of magnitude faster than τ_α_, the two times become comparable for a molecular glass.^14^ Based on a recent dielectric spectroscopy study,^30^ τ_α_ slows down in the cross-linked PEG-X500 at ambient temperature, suggesting ω_B_τ_α_ > 1 at GHz frequencies. Hence, BLS of PEG-X500 should measure the high frequency *M*_∞_ (Figure 4), provided that the α-relaxation is also probed by BLS. This conjecture is examined next.

**Figure 5:**
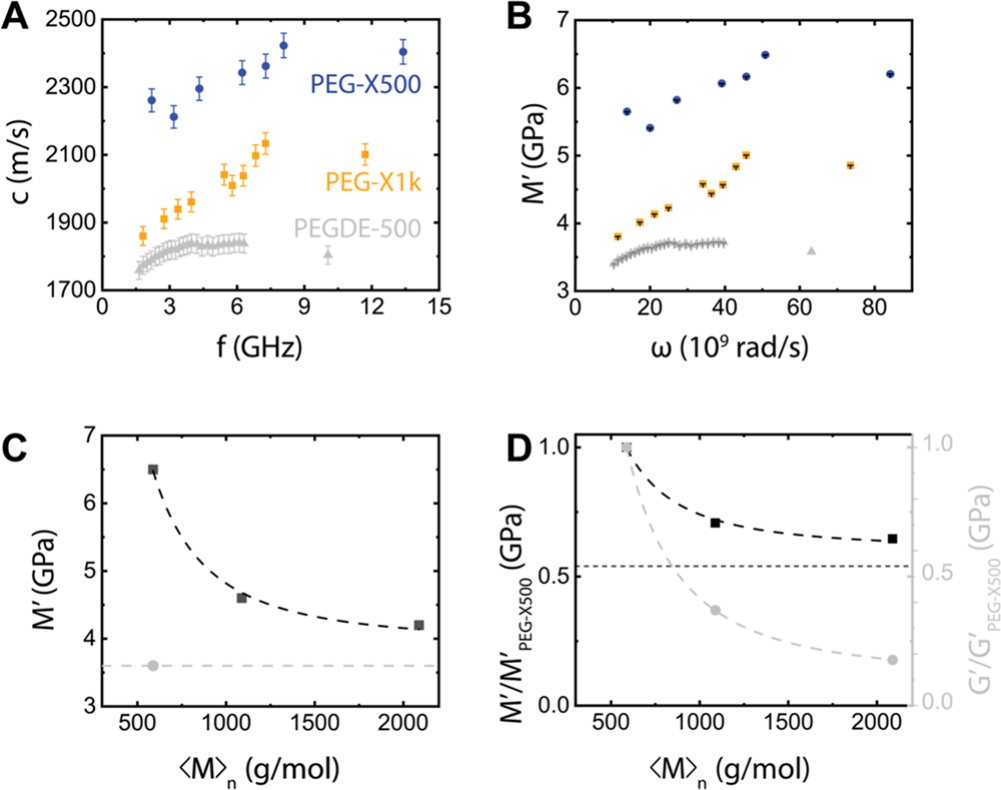
Dispersion of (A) the sound velocity, c (*f*_B_) and (B) longitudinal modulus *M*′ (ω_B_) for the three indicated PEG systems with ω_B_ = 2π*f*_B_. (C) *M*′ at 25 °C as a function of the molar mass of PEGDE. Gray circle corresponds to un-cross-linked precursor PEGDE-500. Dashed lines are guides to the eye. (D) Normalized longitudinal (*M*′, black) and shear (*G*′, gray) moduli to the corresponding values of PEG-X500 as a function of the molar mass of PEGDE plus DAB. The dashed horizontal line at 0.54 indicates the normalized *M*′ ratio of PEGDE-500.

The *q*-dependence of the BLS spectrum is studied at 25 °C for the PEGDE-500 precursor and two cross-linked (PEG-X500 and PEG-X1k) films. From the raw experimental BLS spectra (Figure S6), the dispersions *c(*ω) and *M*′(ω) are calculated and shown in Figure 5, including the values obtained at the highest frequency in backscattering mode. The dispersion *c*(*q*) and dissipation (sound absorption) Γ/*q*^2^(*q*) for the same samples are shown in Figure S7. Several observations emerge from Figure 5: (i) The comparison of *M′* among the different networks indicates frequency independence above 8 GHz (Figure 5A), and the plateau *M*′_∞_ is reached at ω_B_ > 50 × 10^9^ rad/s (Figure 5B); (ii) unexpectedly, *M′*(ω) for PEGDE-500 (Figure 5A,B, gray points) reaches its elastic limit *M*′_∞_ at lower frequencies (ω_B_ ≥ 25 × 10^9^ rad/s) compared to the cross-linked PEG-X500 (ω_B_ ≥ 50 × 10^9^ rad/s); (iii) the high-frequency loss factor  (see eq 2b) is higher (Figure S7b) compared to its low-frequency counterpart  (Figure 2); (iv) Based on the drop of the sound velocity with temperature, *M*′_∞_ (eq 2a) decreases with increasing temperature, in contrast to virtually temperature independent *G*′.^4^

Each one of these observations is elaborated below.

**Figure 6:**
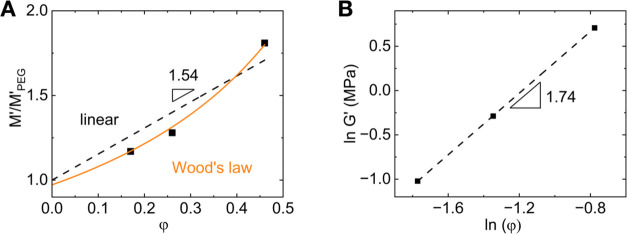
(A) Plot of *M*′ (φ) at 25 °C according to linear (black dashed line), corresponding to *M*′*_n_*/*M*′_*o*_ = 2.5 and Wood’s rule of mixtures (orange solid line) corresponding to *M*′*_n_*/*M*′_*o*_ = 20. Lines are fits under the constraint *M*′/ *M*′_PEG_ (φ = 0) = 1. (B) Logarithmic plot of *G*′ (at 65 °C) vs φ resulting in *G*′ ∼ φ^1.7^.

(i) Comparison of moduli: Based on Figure 5A,B, the experimental *M*′(ω) values obtained at the 90A geometry (*f* ≥ 8 GHz or ω_B_ > 50 × 10^9^ rad/s) represent *M*′_∞_ revealing cross-linking effect, the longitudinal moduli ratio *M*′_∞,sPEG-X500_/*M*′_∞,PEG-X1k_ ≃ 1.4, which is 50% that of for the corresponding shear moduli ratio, *G*′_PEG-X500_/*G*″ _PEG-X1k_ ≃ 2.7 at low frequencies (ω < 100 rad/s). For PEG-X500, *G*′ = 2.03 MPa (Figure 2) is about 4000 times smaller than *M*′. At GHz frequencies, however, , as discussed above, since the high-frequency limiting value of *G*′_∞_ should be in the GPa range (Figure 4).
  Theoretically, the BLS signal in swollen polymeric gels arises from the density fluctuations that propagate through the fluid and the elastic network with volume fraction φ.^15^ In the case of strong frictional damping (γ) due to the fluid within the network, and finite coupling κ of the elastic waves between the fluid and the network, a single longitudinal acoustic phonon is observed with an effective sound velocity *c* and *M*′_eff_, hence *M*′ values lie between the respective values in the fluid (*M*′*_o_*) and “pure network” *M*′*_n_* (eq 3)
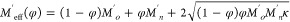
3a
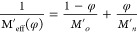
3bFor polyacrylamide hydrogels and solutions, the polymer volume fraction φ was reported to be the dominant factor controlling the *M*′ value^8^ and *M′*_eff_ (φ) was found to follow Wood’s rule (eq 3b) with constant *M′_o_* (for water) and *M′_n_* (bulk polyacrylamide). Earlier experiments on *M′* of poly(vinyl alcohol) and poly(vinyl chloride) gels in water and acetone, respectively favor the linear rule of mixtures, however, with a φ-dependent *M_n_*.^40^ We adopt this analysis for the present dry networks, by considering that the dangling ends and disperse strands (Figure 1B) effectively constitute the fluid phase due to their fluctuations, with a sound velocity similar to that in the precursor PEGDE. In the case of low coupling (κ ≪ 1), the effective *M*′_eff_ of eq 3 follows the linear rule of mixtures, suggesting that the longitudinal modulus of the fluid (*M*_o_) and network (*M_n_*) are linearly weighted to φ.
  To assess the chemical compatibility of PEG strands in comparison to the cross-linked areas arising from the DAB and the epoxide reaction, we employ the Hoftyzer–van Krevelen method (Supporting Information) on solubility parameter which offers two main insights. First, that even though between the uncross-linked hydrophobic DAB and hydrophilic PEG units exists a large solubility difference (Table S4), this difference is reversed upon cross-linking which forces incompatible species to coexist. The cross-linked region, despite the presence of DAB with –(CH_2_)_4_– groups of lower solubility, has a higher eventual solubility due to the exposed −OH groups. Second, a comparison of the three networks PEG-X500, X1k and X2k, exhibits an overall solubility variation <10% despite the larger hydrophilic contribution of the more numerous ethylene oxide groups at PEG-X2k compared to PEG-X500, and PEG dangling ends are not chemically incompatible with the cross-linked network. Therefore, overall network solubility and chemical compatibility cannot account for differences observed among networks.
  Under the strong assumption that the investigated samples are structurally homogeneous dry networks with average molar mass of strands between cross-links *M*_c,phantom_, their cross-link fraction φ can be approximated as the volume fraction of these strands. Hence, for the networks, φ ≃ *N*_c_*b*^3^/ξ^3^, where *N*_c_ = (*M*_c,phantom_ – 88.15)/*M*_m_ (*M*_m_ = 137 g/mol is the Kuhn molar mass of the ethylene oxide monomer and 88.15 g/mol is the molar mas of DAB) and ξ = *bN*_c_^1/2^ (*b* ≃ 0.8 nm is the Kuhn segment length). Based on the *M*_c,phantom_ values of Table 1, φ amounts to 0.45, 0.27, and 0.19 for PEG-X500, PEG-X1k and PEG-X2k, respectively. The linear rule of mixtures (eq 3a for zero coupling, κ = 0) does not adequately represent the experimental *M*′ with *M*′_o_ ≡ *M*′_PEG_ = 3.6 GPa and a constant value of the elasticity contrast, *M*′*_n_*/*M*′*_o_* ≈ 2.5 (black dashed line in Figure 6A). On the other hand, Wood’s rule provides a better representation of *M*′(φ), however, with an unrealistically high ratio *M*′*_n_*/*M*′_*o*_ ≈ 20 (red dashed line in Figure 6A). For finite elastic waves coupling constant κ ≤ 1, eq 3 leads to an unrealistic ratio *M′_n_*/*M*′*_o_* < 1, as well. In this context, we should note that based on the estimated *M*_c,phantom_ from shear rheometry (Figure 2 and Table 1), ξ is about 1.7 and 2.8 nm for PEG-X500 and PEG-X1k, respectively. In the absence of an experimental verification of ξ by SAXS, the elasticity enhancement can be rationalized by the increase of the effective φ discussed above. Since the estimation of the cross-link fraction φ is highly approximate, the representation of *M*′_eff_ (φ) by the two rules of mixtures is delicate and slightly ambiguous. In fact, the ratios *M*′_PEG-X500_/*M*′_PEG-X1k_ and *M*′_PEG-X500_/*M*′_PEG-X2k_ and the corresponding φ ratios conform better to Wood’s rule than to the linear rule of mixtures (Figure S8). In addition, the limited experimental data on moduli ratios *G*′_PEG-X500_/*G*′ _PEG-X1k_ and *G*′_PEG-X500_/*G*′_PEG-X2k_ conform to the physically meaningful scaling *G*′ ∼ φ^2^ (see Figure S8).^41^ These comparisons support the scaling φ ∼ *N*_c_^1/2^. Nevertheless, the overall analysis should be considered with caution.
(ii) Fast relaxation of PEG-X500: Given the τ_α_ values estimated from the dielectric spectroscopy,^3^ the dispersion in Figure 5A is not due to the α-relaxation process, since ω_B_τ_α_ ≫ 1. In other words, the observed relaxation in PEG500 at GHz frequencies is faster than the α-relaxation process, as also reported for other amorphous polymers.^13^ This conclusion is further supported by the presence of hypersonic dispersion in PEG-X500, which possesses an order of magnitude slower τ_α_ than in PEG-500, also in agreement with the small increase of *T*_g_ upon cross-linking.^3^ According to Figure 5A, the dispersion is shifted to higher frequencies in PEG-X500, suggesting faster relaxation process than in the precursor PEGDE-500, which is an opposite trend to the α-relaxation process. The origin of this fast (subsegmental) relaxation is attributed to the response of PEG segments linked with DAB cross-linkers.
(iii) Loss factor: The phonon absorption Γ/q^2^ (Figure S7b) increases with the cross-link density and becomes stronger for PEG-X500 only at low *q*’s (<0.01 nm^–1^), unlike the dispersion of *c*(*q*) (Figure S7a). This finding suggests that the increased phonon propagation at long wavelengths is the result of phonon scattering from network heterogeneities (that make the density nonuniform). In this context, the high-frequency loss factor tan(δ) = Γ_B_/ω_B_ (Figure S90b) for M* is higher (by about 10%) compared to the respective low-frequency ratio *G*″(ω)/*G*′(ω). This reflects different effects of network heterogeneities at different length scales. Based on the theory of BLS from polymer gels discussed above, in the case of strong frictional damping on the elastic network resulting from the fluid moving relative to the network (strong friction limit) and high frequencies (ω_B_τ ≫ 1)^15^
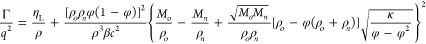
4awhere η_L_ is the longitudinal viscosity and subscripts “*o*” and “*n*” denote the fluid and pure polymeric network, respectively.
  At low volume fractions φ, if we assume similar densities and elasticity of fluid and network (ρ*_o_* ≈ ρ*_n_* and *M_o_* ≈ *M_n_*), eq 4a simplifies to
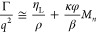
4beq 4b suggests an additive contribution of the longitudinal viscosity η_L_ and the network to the pure fluid dissipation. According to Figure S9a, the experimental Γ/*q*^2^ increases with the cross-link volume fraction (or inverse molar mass of strand between cross-links) at low frequencies or large phonon wavelengths (Figure S7b). In eq 4a, the network contribution is -like the fluid dissipation- proportional to *q*^2^ or ω^2^, hence Γ/*q*^2^ is frequency-independent. However, in the case of small damping in the fluid, a *q*-independent frictional damping  is added to the fluid dissipation (η_L_*q*^2^/ρ*_o_*).^15^ In the latter case, the phonon absorption Γ/*q*^2^ increases strongly at low *q*’s, as observed experimentally, while it asymptotically assumes a φ-independent fluid-like value at high *q*’s (Figure S7b).
(iv) Temperature dependence: The different mechanisms associated with low frequency *G*′ and high frequency *M*′ are also evidenced from their temperature dependence. While the former is virtually temperature independent, the latter decreases by about 50% with increasing temperature from 20 to 60 °C for PEG-X500 (Figure 3). We recall, however, that while both moduli depend on the fraction of cross-links, *G*′ reflects the entropic elasticity of the entire network (*G*′ = 0 for fluids) and is generally described in the context of rubber elasticity theory *M*′ is a local (small scale) property and is described to a first approach by considering the network as an effective binary system (where the fluid part reflects dangling ends, loops, defects). In fact, for the precursor PEGDE-500, the softening of *M*′ is about 40% over the same temperature range and is mainly due to packing (density). On the other hand, the phonon absorption Γ/*q*^2^ and tan δ at *q*_||_ = 0.0167 nm^–1^ decreases by about 20% for PEG-X500, while it is virtually constant for its precursor PEGDE-500 (and for PEGDE-X1k). This finding implies different mechanisms for the propagation and dissipation of hypersonic waves and hence *M*′ and *M*″, respectively. Since the phonon absorption relates to the longitudinal viscosity, ρΓ_B_/*q*^2^= η_L_(ω), the heterogeneous cross-linking seems to introduce an additional low frequency contribution (Figure S9a), which is stronger for PEG-X500. On the other hand, the high frequency limit of η_L_(ω > 60 × 10^9^ rad/s) is independent of cross-linking and controlled by the fast dynamics of PEG segments between cross-links.

## Conclusions

We have measured the shear moduli G*(ω) by conventional and piezo-rheology and the longitudinal moduli M*(ω) by Brillouin light spectroscopy (BLS) for three solvent-free epoxy networks prepared from linear PEG with number average molar masses of 500, 1000, and 2000 g/mol, and cross-linked with a flexible tetrafunctional diamine. The key result of this work is the ability to provide reliable data for the network elasticity at different frequency regimes, hence different length scales: low (rheology), high (piezo rheology) and ultrahigh (BLS). The importance of this set of data is elaborated next by summarizing the key findings.

The ratio of the frequency independent *G*′ (ω < 10^4^ rad/s) from rheology at two cross-link volume fractions is *G*′(φ = 0.45)/ *G*′(φ = 0.19) ≃ 5.6. The respective ratio of *M*′_∞_ from BLS at backscattering is *M*′_∞_(φ = 0.45)/ *M*′_∞_(φ = 0.19) ≃1.6, i.e., much weaker than for *G′*. This difference is rationalized in the context of the phantom network model for *G*′ *∼N*_c_^–1^ ∼ φ^2^ (*N*_c_ being the number of segments between cross-links) and Wood’s rule for mixtures (inverse dependence of *M*′ on φ), by considering the heterogeneous dry networks are binary systems comprising segments between cross-links and dangling ends of loops. However, a BLS theory of polymer hydrogels favors a linear rule of mixtures in the case of slow relaxation (ω_B_τ ≫ 1) at GHz frequencies and negligibly small coupling in a fluid-network mixture.^15^ In the same theoretical framework, the phonon absorption Γ/q^2^ should be robust to *q*-variation, in contrast to the experimental behavior. Conversely, the present experimental data with dry cross-linked polymer networks favors a q-independent frictional damping to the fluid dissipation. For these networks, a hypersonic dissipation due to structural relaxation is found to be faster compared to the uncross-linked precursor. The experimental evidence presented suggests that the correlation between *G*′ at low frequency and *M*′ at high frequency depends on the molecular details of the network (cross-link fraction and distribution, defects, heterogeneities). It seems that the two moduli of the dry networks are correlated via the cross-link fraction (Figure 6) as proposed for hydrogels recently.^8^ However, more data with different networks and cross-link densities would be needed to establish this analogy, also with different chemistries are needed in order to provide deeper insights toward the development of rigorous theory for the elastic *M*′. These results would hopefully trigger more experimental efforts in this important direction.

## Supporting Information Available

Table of mixing compositions; refractive indices of precursors; DSC of 2k precursor and network; additional BLS spectra at 25 and 60 °C; *q*-dependence of BLS spectra; phonon absorption and tan(δ); SAXS of networks (PDF)

## References

[ref1] Vining K. H.; Mooney D. J. Mechanical Forces Direct Stem Cell Behaviour in Development and Regeneration. Nat. Rev. Mol. Cell Biol. 2017, 18 (12), 728–742. 10.1038/nrm.2017.108. 29115301 PMC5803560

[ref2] Northcott J. M.; Dean I. S.; Mouw J. K.; Weaver V. M. Feeling Stress: The Mechanics of Cancer Progression and Aggression. Front. Cell Dev. Biol. 2018, 6, 17 10.3389/fcell.2018.00017. 29541636 PMC5835517

[ref3] Rubinstein M.; Colby R. H. Polymer Physics; Oxford University Press, 2003.

[ref4] Li B.; Alexandris S.; Pantazidis C.; Moghimi E.; Sakellariou G.; Vlassopoulos D.; Filippidi E. Mechanical Properties of Epoxy Networks with Metal Coordination Bonds: Insights from Temperature and Molar Mass Variation. Macromolecules 2024, 57 (19), 9088–9096. 10.1021/acs.macromol.4c01143. 39399830 PMC11468226

[ref5] Schroyen B.; Vlassopoulos D.; Puyvelde P. V.; Vermant J. Bulk Rheometry at High Frequencies: A Review of Experimental Approaches. Rheol. Acta 2020, 59 (1), 1–22. 10.1007/s00397-019-01172-w.

[ref6] Müller D. J.; Dumitru A. C.; Giudice C. L.; Gaub H. E.; Hinterdorfer P.; Hummer G.; De Yoreo J. J.; Dufrêne Y. F.; Alsteens D. Atomic Force Microscopy-Based Force Spectroscopy and Multiparametric Imaging of Biomolecular and Cellular Systems. Chem. Rev. 2021, 121 (19), 11701–11725. 10.1021/acs.chemrev.0c00617. 33166471

[ref7] Kennedy B. F.; Wijesinghe P.; Sampson D. D. The Emergence of Optical Elastography in Biomedicine. Nat. Photonics 2017, 11 (4), 215–221. 10.1038/nphoton.2017.6.

[ref8] Rodríguez-López R.; Wang Z.; Oda H.; Erdi M.; Kofinas P.; Fytas G.; Scarcelli G. Network Viscoelasticity from Brillouin Spectroscopy. Biomacromolecules 2024, 25 (2), 955–963. 10.1021/acs.biomac.3c01073. 38156622 PMC10865340

[ref9] Scarcelli G.; Polacheck W. J.; Nia H. T.; Patel K.; Grodzinsky A. J.; Kamm R. D.; Yun S. H. Noncontact Three-Dimensional Mapping of Intracellular Hydromechanical Properties by Brillouin Microscopy. Nat. Methods 2015, 12 (12), 1132–1134. 10.1038/nmeth.3616. 26436482 PMC4666809

[ref10] Palombo F.; Fioretto D. Brillouin Light Scattering: Applications in Biomedical Sciences. Chem. Rev. 2019, 119 (13), 7833–7847. 10.1021/acs.chemrev.9b00019. 31042024 PMC6624783

[ref11] Bailey M.; Alunni-Cardinali M.; Correa N.; Caponi S.; Holsgrove T.; Barr H.; Stone N.; Winlove C. P.; Fioretto D.; Palombo F. Viscoelastic Properties of Biopolymer Hydrogels Determined by Brillouin Spectroscopy: A Probe of Tissue Micromechanics. Sci. Adv. 2020, 6 (44), eabc1937 10.1126/sciadv.abc1937. 33127678 PMC7608813

[ref12] Adichtchev S. V.; Karpegina Y. A.; Okotrub K. A.; Surovtseva M. A.; Zykova V. A.; Surovtsev N. V. Brillouin Spectroscopy of Biorelevant Fluids in Relation to Viscosity and Solute Concentration. Phys. Rev. E 2019, 99 (6), 062410 10.1103/PhysRevE.99.062410. 31330595

[ref13] Hecksher T.; Torchinsky D. H.; Klieber C.; Johnson J. A.; Dyre J. C.; Nelson K. A. Toward Broadband Mechanical Spectroscopy. Proc. Natl. Acad. Sci. U.S.A. 2017, 114 (33), 8710–8715. 10.1073/pnas.1707251114. 28765374 PMC5565458

[ref14] Voudouris P.; Gomopoulos N.; Grand A. L.; Hadjichristidis N.; Floudas G.; Ediger M. D.; Fytas G. Does Brillouin Light Scattering Probe the Primary Glass Transition Process at Temperatures Well above Glass Transition?. J. Chem. Phys. 2010, 132 (7), 074906 10.1063/1.3319687. 20170250

[ref15] Marqusee J. A.; Deutch J. M. Brillouin Light Scattering from Polymer Gels. J. Chem. Phys. 1981, 75 (11), 5239–5245. 10.1063/1.441988.

[ref16] Aldridge M.; Wineman A.; Waas A.; Kieffer J. In Situ Analysis of the Relationship between Cure Kinetics and the Mechanical Modulus of an Epoxy Resin. Macromolecules 2014, 47 (23), 8368–8376. 10.1021/ma501441c.

[ref17] Kallivokas S. V.; Sgouros A. P.; Theodorou D. N. Molecular Dynamics Simulations of EPON-862/DETDA Epoxy Networks: Structure, Topology, Elastic Constants, and Local Dynamics. Soft Matter 2019, 15 (4), 721–733. 10.1039/C8SM02071J. 30629083

[ref18] Athanasiou T.; Auernhammer G. K.; Vlassopoulos D.; Petekidis G. A High-Frequency Piezoelectric Rheometer with Validation of the Loss Angle Measuring Loop: Application to Polymer Melts and Colloidal Glasses. Rheol. Acta 2019, 58 (9), 619–637. 10.1007/s00397-019-01163-x.

[ref19] Athanasiou T.; Geri M.; Roose P.; McKinley G. H.; Petekidis G. High-Frequency Optimally Windowed Chirp Rheometry for Rapidly Evolving Viscoelastic Materials: Application to a Crosslinking Thermoset. J. Rheol. 2024, 68 (3), 445–462. 10.1122/8.0000793.

[ref20] Furst E. M.; Squires T. M. Microrheology, 1st ed.; Oxford University Press: Oxford, 2017.

[ref21] Wenzel J.; Ruprich N.; Sperlich K.; Stachs O.; Schunemann M.; Leyh C.; Kalies S.; Heisterkamp A. Non-Invasive Full Rheological Characterization via Combined Speckle and Brillouin Microscopy. IEEE Access 2022, 10, 75527–75535. 10.1109/ACCESS.2022.3192463.

[ref22] Zhong M.; Wang R.; Kawamoto K.; Olsen B. D.; Johnson J. A. Quantifying the Impact of Molecular Defects on Polymer Network Elasticity. Science 2016, 353 (6305), 1264–1268. 10.1126/science.aag0184. 27634530

[ref23] Lin T. S.; Wang R.; Johnson J. A.; Olsen B. D. Revisiting the Elasticity Theory for Real Gaussian Phantom Networks. Macromolecules 2019, 52 (4), 1685–1694. 10.1021/acs.macromol.8b01676.

[ref24] Morsch S.; Liu Y.; Lyon S. B.; Gibbon S. R. Insights into Epoxy Network Nanostructural Heterogeneity Using. *ACS*. ACS Appl. Mater. Interfaces 2016, 8, 959–966. 10.1021/acsami.5b10767. 26694687

[ref25] Nguyen H. K.; Aoki M.; Liang X.; Yamamoto S.; Tanaka K.; Nakajima K. Local Mechanical Properties of Heterogeneous Nanostructures Developed in a Cured Epoxy Network: Implications for Innovative Adhesion Technology. ACS Appl. Nano Mater. 2021, 4 (11), 12188–12196. 10.1021/acsanm.1c02692.

[ref26] Haba D.; Kaufmann J.; Brunner A. J.; Resch K.; Teichert C. Observation of Elastic Modulus Inhomogeneities in Thermosetting Epoxies Using AFM e Discerning Facts and Artifacts. Polymer 2014, 55, 4032–4040. 10.1016/j.polymer.2014.06.030.

[ref27] Sahagun C. M.; Knauer K. M.; Morgan S. E. Molecular Network Development and Evolution of Nanoscale Morphology in an Epoxy-Amine Thermoset Polymer. J. Appl. Polym. Sci. 2012, 126 (4), 1394–1405. 10.1002/app.36763.

[ref28] Saalwächter K.; Ziegler P.; Spyckerelle O.; Haidar B.; Vidal A.; Sommer J. U. 1H Multiple-Quantum Nuclear Magnetic Resonance Investigations of Molecular Order Distributions in Poly(Dimethylsiloxane) Networks: Evidence for a Linear Mixing Law in Bimodal Systems. J. Chem. Phys. 2003, 119 (6), 3468–3482. 10.1063/1.1589000.

[ref29] Saalwächter K. Proton Multiple-Quantum NMR for the Study of Chain Dynamics and Structural Constraints in Polymeric Soft Materials. Prog. Nucl. Magn. Reson. Spectrosc. 2007, 51 (1), 1–35. 10.1016/j.pnmrs.2007.01.001.

[ref30] Spyridakou M.; Iliopoulou E.; Peponaki K.; Alexandris S.; Filippidi E.; Floudas G. Heterogeneous Local Dynamics in Mussel-Inspired Elastomers. Macromolecules 2023, 56 (11), 4336–4345. 10.1021/acs.macromol.3c00356.

[ref31] Patterson G. D.; Latham J. Hypersonic Relaxation in Poly(Ethylene Oxide). Macromolecules 1977, 10 (6), 1414–1415. 10.1021/ma60060a049.

[ref32] Wang C. H.; Lin Y. H.; Jones D. R. Brillouin Scattering and Segmental Motion of a Polymeric Liquid, II. Mol. Phys. 1979, 37 (1), 287–298. 10.1080/00268977900100241.

[ref33] Fytas G.; Lin Y. H.; Chu B. Rayleigh-Brillouin Spectra of Polymer Fluids: Polyphenylmethyl Siloxane and Polydimethylphenylmethyl Siloxane. J. Chem. Phys. 1981, 74 (6), 3131–3138. 10.1063/1.441523.

[ref34] Matsukawa M.; Yamura H.; Nakayama S.; Otani T. Brillouin Scattering Study of Epoxy Adhesive Layers during Cure. Ultrasonics 2000, 38 (1), 466–469. 10.1016/S0041-624X(99)00198-5. 10829706

[ref35] Alig I.; Lellinger D.; Nancke K.; Rizos A.; Fytas G. Dynamic Light Scattering and Ultrasonic Investigations during the Cure Reaction of an Epoxy Resin. J. Appl. Polym. Sci. 1992, 44 (5), 829–835. 10.1002/app.1992.070440510.

[ref36] Floudas G.; Fytas G.; Alig I. Brillouin Scattering from Bulk Polybutadiene: Distribution of Relaxation Times versus Single Relaxation Time Approach. Polymer 1991, 32 (13), 2307–2311. 10.1016/0032-3861(91)90065-Q.

[ref37] Comez L.; Fioretto D.; Scarponi F.; Monaco G. Density Fluctuations in the Intermediate Glass-Former Glycerol: A Brillouin Light Scattering Study. J. Chem. Phys. 2003, 119 (12), 6032–6043. 10.1063/1.1601608.

[ref38] Santucci S. C.; Fioretto D.; Comez L.; Gessini A.; Masciovecchio C. Is There Any Fast Sound in Water?. Phys. Rev. Lett. 2006, 97 (22), 225701 10.1103/PhysRevLett.97.225701. 17155812

[ref39] O’Steen B. L.; Wang C. H.; Fytas G. Rayleigh-Brillouin Scattering Studies of the Rotation-Translation Coupling and Bulk Viscosity Relaxation of Liquids Composed of Anisotropic Molecules: P-Anisaldehyde and Aniline. J. Chem. Phys. 1984, 80 (8), 3774–3780. 10.1063/1.447156.

[ref40] Ng S. C.; Li Y. Brillouin Light-Scattering from Polymer Gels. J. Phys. II 1993, 3 (8), 1241–1245. 10.1051/jp2:1993194.

[ref41] Graessley W. W. Polymeric Liquids & Networks: Structure and Properties, 1st ed.; Garland Science: New York, 2003.

