# Supplementary material for: Multiscale Elasticity of Epoxy Networks by Rheology and Brillouin Light Spectroscopy
Source: J Phys Chem B. 2024 Dec 4;128(50):12628–37. doi: 10.1021/acs.jpcb.4c06492 (PMC11664584; doi:10.1021/acs.jpcb.4c06492)
Supplement: Supplementary file 1 — jp4c06492_si_001.pdf [file jp4c06492_si_001.pdf]

Supplementary Information

Multiscale Elasticity of Epoxy Networks by Rheology and Brillouin Light Spectroscopy

Emmanouela Filippidi[1,2], Anuj K. Dhiman[3], Benke Li[2], Thanasis Athanasiou[2], Dimitris Vlassopoulos[1,2], George Fytas[2,4*]*

1. Department of Materials Science and Engineering, University of Crete, Heraklion, 70013, Greece

2. Institute of Electronic Structure and Laser, FORTH, Heraklion, 70013, Greece

3. Faculty of Physics, Adam Mickiewicz University, Uniwersytetu Poznanskiego 2, Poznan, 61614, Poland

4. Max Planck Institute for Polymer Research, Ackermannweg 10, Mainz, 55128, Germany

*fytas@mpip-mainz.mpg.de
*filippidi@materials.uoc.gr

Contents

Table S1. Table of mixing compositions for network formation, glass transition and melting temperatures and table of refractive indices of melt precursors

Table S2. Refractive indices of precursor polymers

Figure S1. DSC of PEGDE-2k and PEGX-2k

Figure S2. Small angle X-ray scattering of PEG-X500 and PEG-X1k

Figures S3-S4. BLS spectra of PEG-500 and PEG-X1k in 90A transmission geometry

Figure S5. BLS spectra of PEGDE-500 and PEGDE-2k at 60°C

Figure S6. *q*-dependence of BLS spectra of PEGDE-500, PEG-X500 and PEG-X1k

Figure S7. Phonon dispersion, sound absorption of PEGDE-500, PEG-X500 and PEG-X1k

Figure S8. Estimates of the volume fraction ratio

Figure S9. Sound absorption and tan(δ) of PEGDE-500, PEG-X500 and PEG-X1k

Tables S3-S5. Calculation of solubility parameters, δ, via the Hoftyzer - Van Krevelen method

Table S1. Table of mixing compositions (% mass) for network formation, and networks' glass transition and melting temperatures

network	polymer PEGDE	crosslinker DAB	T_g (°C)	T_m (°C)
	mole ratio PEGDE : DAB = 1.00 : 0.50			
**PEG-X500**	91.9	8.1	-40.6	—
**PEG-X1k**	95.78	4.2	-50.2	24
**PEG-X2k**	97.84	2.2	not identified	37

Table S2. Refractive indices of precursor polymers

precursor	temperature (°C)	refractive index (± 0.0001)
PEGDE-500	30	1.4677
	60	1.4629
	65	1.4622
PEGDE-1k	60	1.4647
	65	1.4642

DSC of PEGDE-2k and PEGX-2k

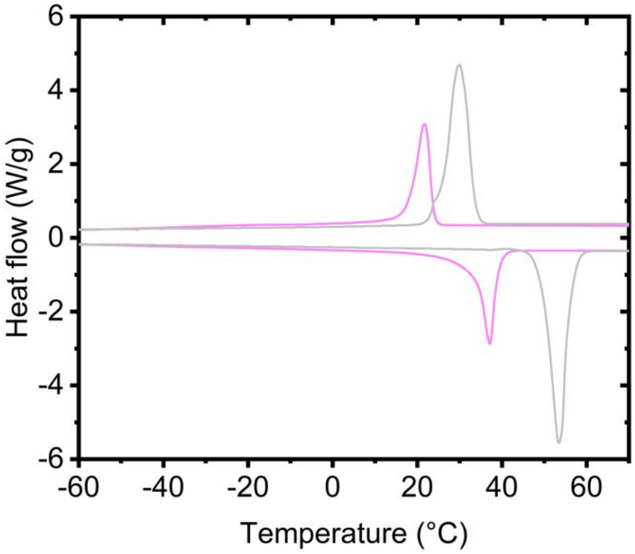

Figure S1. Differential scanning calorimetry (DSC) of the PEGDE-2k melt (gray) and PEG-X2k network (magenta). Peak crystallization temperatures: 30 ˚C for the melt (gray) and 21.7 ˚C for the PEG-X2k. Melting temperatures: 37 ˚C (for the PEGX-2k) and 53.4 ˚C for the melt (gray).

Small angle X-ray scattering of PEG-X500 and PEG-X1k

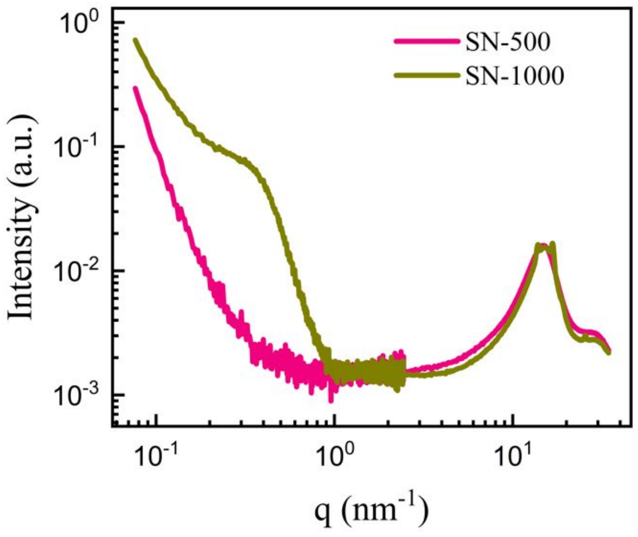

Figure S2. Small angle X-ray scattering of PEG-X500 (pink) and PEG-X1k (olive). For PEG-X500, lack of features beyond the PEO amorphous peak at ~15nm^{-1} do not support any structure formation. For PEG-X1k signs of crystallinity develop at the wide-angle regime (~15 nm^{-1}), accompanied by a crystallinity-linked (i.e. temperature reversible) shoulder at 0.4 nm^{-1} (corresponding to ~16 nm). There is no evidence of any density correlation peak in the range 1 to 1.5 nm^{-1}.

BLS spectra of PEG-500 and PEG-X1k in 90A transmission geometry

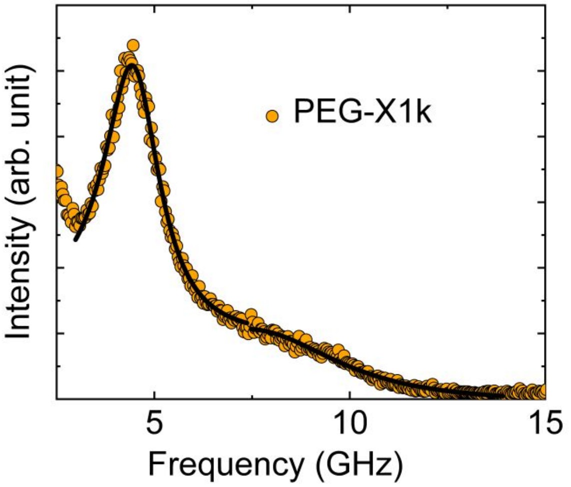

Figure S3. BLS spectra of PEG-X1k at 60 °C recorded in the 90A transmission geometry ($q_{||} = 0.0167\ nm^{-1}$). The phonon frequency, $f_B = 4.42\ GHz$, leads to the longitudinal sound velocity 1660 *m/s*. The broad peak at high frequencies is the contribution of the artificial backscattering.

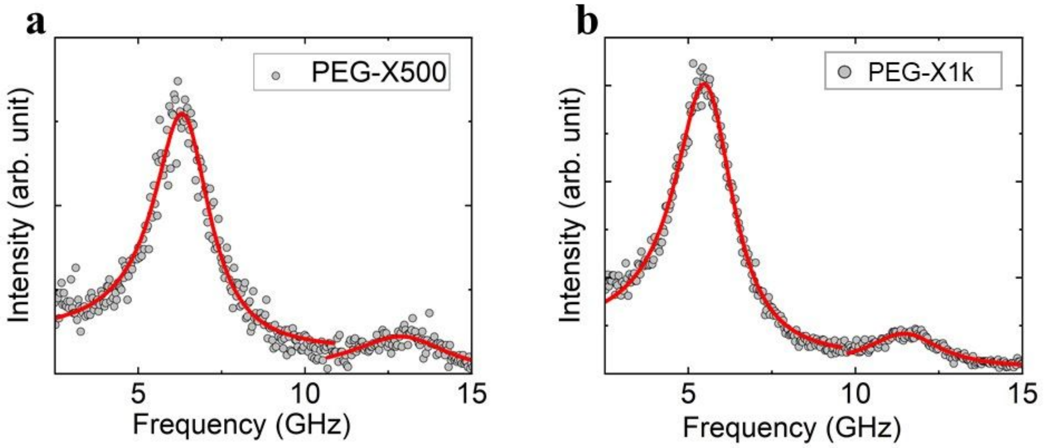

Figure S4. **BLS spectra (anti-Stokes side)** of PEG-X500 and PEG-X1k at 25˚C recorded at 90A geometry ($q_{||} = 0.0167\ nm^{-1}$). The second peak at high frequencies is the contribution of the artificial backscattering.

BLS spectra of PEGDE-500 and PEGDE-2k at 60 °C

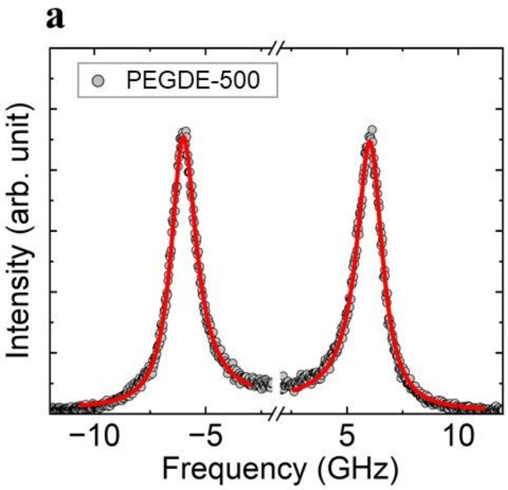

Figure S5. **BLS spectra** recorded at 60˚C of (A) PEGDE-500 (phonon frequency $f_B = 6.0$ *GHz*, $\Gamma_B = 1.4$ *GHz*, tan(δ) = 0.23) and PEGDE-2k ($f_B = 4.07$ *GHz*, $\Gamma_B = 0.9$ *GHz*, tan(δ) = 0.22) at 90˚ (in bulk, $q = 0.0245$ nm^{-1}, $n = 1.47$) and 90A transmission ($q_{||} = 0.0167$nm^{-1}), respectively. The high frequency peak in (b) is the contribution from the artificial backscattering. Both materials display the same longitudinal sound velocity (c= 1530 ± 15 *m/s*) within experimental error.

***q*-dependence of BLS spectra of PEGDE-500, PEG-X500 and PEG-X1k**

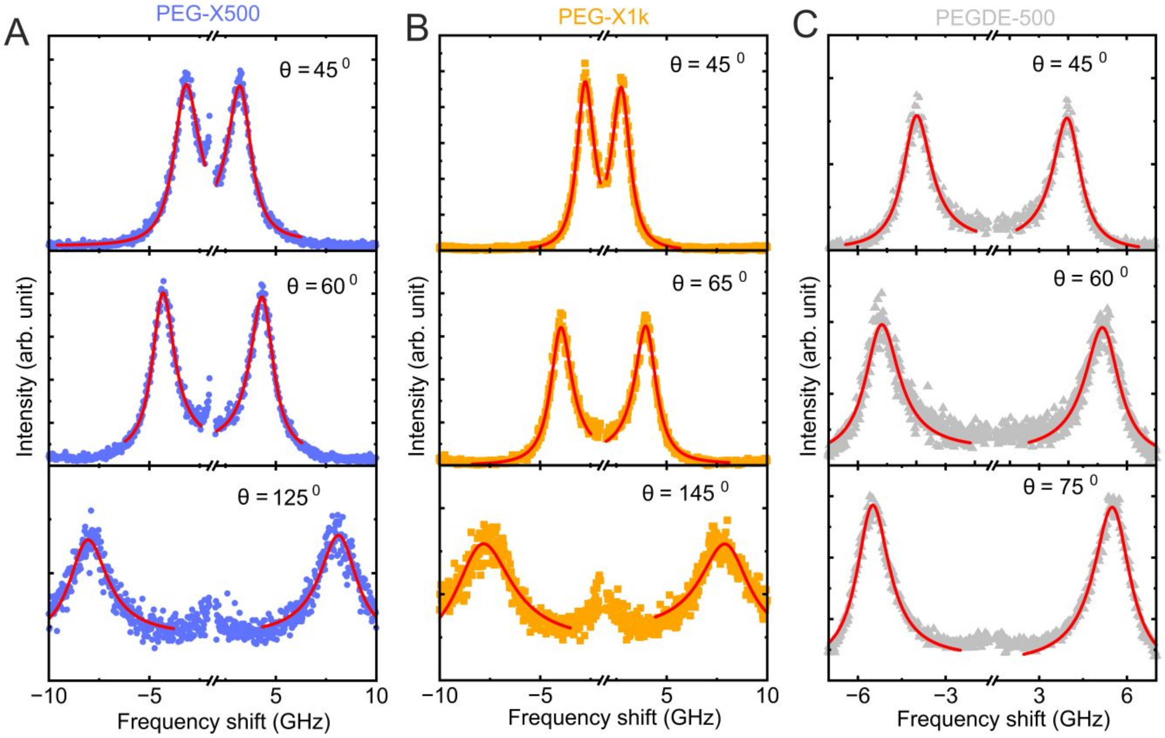

Figure S6. *q*-dependence of BLS spectra at 25 °C. Variation of the BLS spectra with scattering angle, θ, for (A) PEG-X500, (B) PEG-X1k films with $q_{||} = (4\pi/\lambda)\, sin(\theta/2)$ and (C) for PEGDE-500 melt in the liquid state ($q = nq_{||}$, n = 1.48).

Phonon dispersion and sound absorption of PEGDE-500, PEG-X500 and PEG-X1k

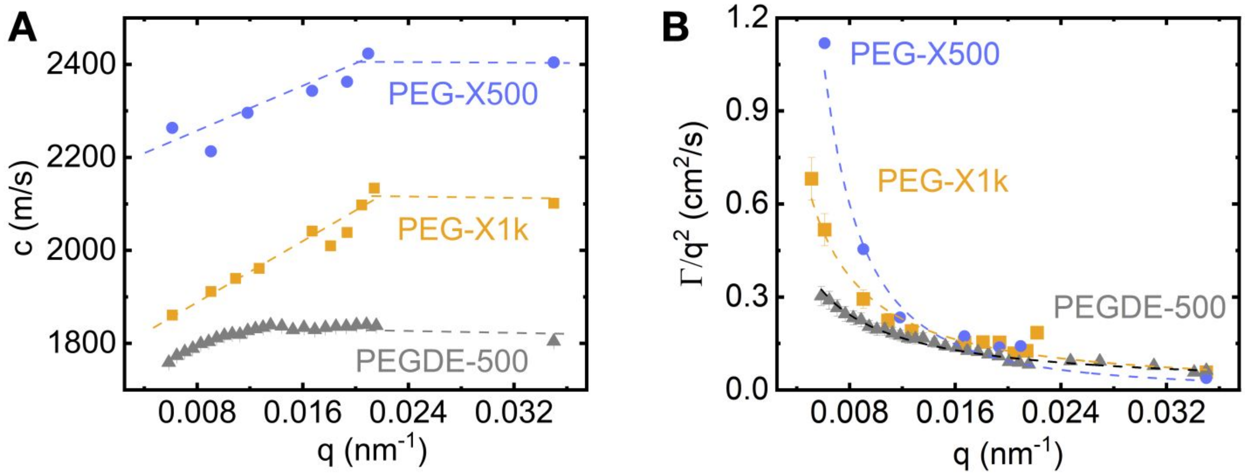

Figure S7. (A) Sound velocity dispersion and (B) phonon absorption of PEGDE-500 precursor, PEG-X500 and PEG-X1k at 25 °C. Lines are guides to the eye.

Estimates of the volume fraction ratio

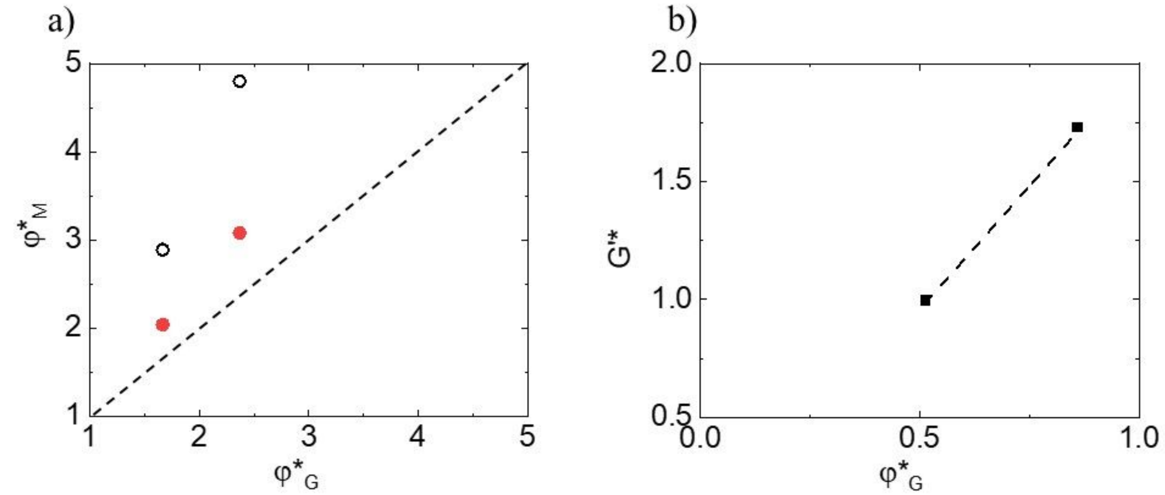

Figure S8. a) The volume fraction ratio, φ_M^*, estimated from the experimental ratio $M'_{\text{PEG-X500}}$/ $M'_{\text{PEG-X1k}}$ and $M'_{\text{PEG-X500}}$/ $M'_{\text{PEG-X2k}}$ according to the linear (open circles) and Wood's (red circles) rule of mixtures vs φ_G^*; $\varphi_G^* = \varphi_{\text{PEG-X500}}/\ \varphi_{\text{PEG-X1k}} = 1.67$ and $\varphi_G^* = \varphi_{\text{PEG-X500}}/\ \varphi_{\text{PEG-X2k}} = 2.37$, where φ in the three samples is computed from N_c, the Kuhn degree of polymerization of strands between

crosslinks (see text). The deviation of the two set of points from of the diagonal is smaller in the case of the Wood's rule. b) The ratio G^{*} = $G'_{PEG\text{-}X500}$/ $G'_{PEG\text{-}X1k}$ to the other two G' for PEG-X1k and PEG-X2k vs φ_G^{*}. The slope conforms to $G^{*} \sim (\varphi^{*})^2$ (see main text).

Sound absorption and tan(δ) of PEGDE-500, PEG-X500 and PEG-X1k

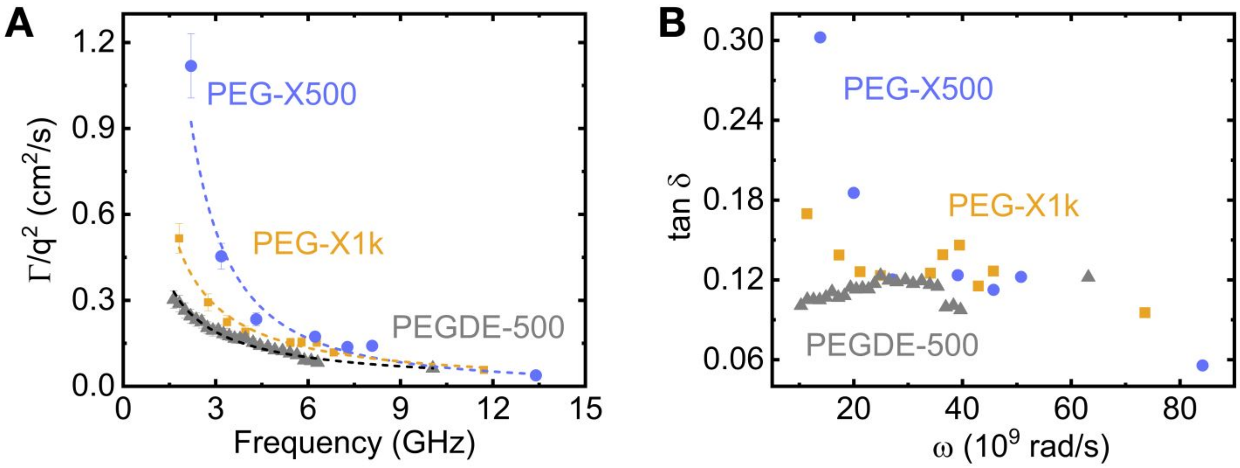

Figure S9. (A) Sound absorption Γ/q^2 and (B) $\tan(\delta) = \Gamma_B/\omega_B$ for the indicated samples at 25 °C.

Calculation of solubility parameters, δ, via the Hoftyzer - Van Krevelen method

We employ the concept of the solubility parameter, δ, following the method of Hoftyzer-van Krevelen,[1] in a similar manner it was performed by Cristiani et al.[2] The solubility parameter

components δ_d, δ_p and δ_h for dispersive, polar and hydrogen bonding, can be calculated from group contributions F_{di} for dispersive, F_{pi} for polar, and E_{hi} for hydrogen bonding forces as follows

$$\delta_d = \frac{\sum F_{di}}{V},\ \delta_p = \frac{\sqrt{\sum F_{pi}^2}}{V},\ \delta_h = \sqrt{\frac{\sum E_{hi}}{V}} \text{ with overall } \delta = \left(\delta_d{}^2 + \delta_p{}^2 + \delta_h{}^2\right)^{1/2}$$

Assuming full epoxy-amine conversion, thus neglecting the presence of -NH2, -NH and epoxide groups, we must consider the structural groups in Table S3.

Table S3. Molar volume and solubility parameter group contributions.

structural group	molar volume V (cm^3/mol)	F_{di} $(MJ/m^3)^{0.5}$/mol	F_{pi} $(MJ/m^3)^{0.5}$/mol	E_{hi} (J/mol)
>N-	12.6	20	800	5000
-CH2-	15.55	270	0	0
>CH-	9.56	80	0	0
-OH	12.45	210	500	20000
-O-	6.45	100	400	3000

Table S4. Molar volume and solubility parameters of the structural units present in PEG-X# networks. The repeating ethylene oxide, EO, of the PEGDE, the butane, BU, of the DAB, and the cross-linker structure group that is constant for all networks, independent of the molar mass of PEGDE.

abbr.	structural group	molar volume V (cm^3/mol)	δ_d	δ_p	δ_h	solubility parameter δ $(MJ/m^3)^{1/2}$
EO	[CH_2-O-CH_2]-	37.55	17.04	10.65	8.94	22.00<br>20.8 from [3]

BU	$-(CH_2)_4-$	62.2	17.363	0	0	17.363
XL	$(OH-CH-CH_2)_2-N-(CH_2)_4$<br>$-N-(CH_2-CH-OH)_2$	237.64	14.139	6.354	19.461	24.88

[3] Literature value from Polymer Properties Database
http://polymerdatabase.com/polymer%20physics/delta%20Table.html

The solubility parameter $\delta_{network} = \delta_{EO}\, x_{EO} + \delta_{XL}\, x_{XL}$, where x_{PEO} and x_{XL} are the mole fractions of EO and XL respectively.

In our networks, 1 mole of XL group, corresponds to n moles of EO groups.

Table S5. Solubility parameter of the entire network for all three networks examined.

network	mol XL	mol EO	$\delta_{network}$ (MPa)$^{1/2}$<br>[second row with EO value 20.8 from x]	$\delta_{network} - \delta_{THF}$<br>(MJ/m^3)$^{1/2}$
PEG-X500	1	8	22.32<br>21.25 (using δ_{EO} = 20.8 from [3])	2.92<br>1.85
PEG-X1k	1	20	22.14<br>20.99 (using δ_{EO} = 20.8 from [3])	2.74<br>1.59
PEG-X2k	1	42	22.07<br>20.89 (using δ_{EO} = 20.8 from [3])	2.67<br>1.49

[3] Literature value from Polymer Properties Database
http://polymerdatabase.com/polymer%20physics/delta%20Table.html

References

(1) van Krevelen, D. W.; Te Nijenhuis, K. *Properties of Polymers*, 4th ed.; Elsevier: Amsterdam, 2009.

(2) Cristiani, T. R.; Filippidi, E.; Behrens, R. L.; Valentine, M. T.; Eisenbach, C. D. Tailoring the Toughness of Elastomers by Incorporating Ionic Cross-Linking. *Macromolecules* **2020**, *53*, 4099–4109. https://doi.org/10.1021/acs.macromol.0c00500.
